# Impact of empagliflozin on subclinical left ventricular dysfunctions and on the mechanisms involved in myocardial disease progression in type 2 diabetes: rationale and design of the EMPA-HEART trial

**DOI:** 10.1186/s12933-017-0615-6

**Published:** 2017-10-12

**Authors:** Andrea Natali, Lorenzo Nesti, Iacopo Fabiani, Enrico Calogero, Vitantonio Di Bello

**Affiliations:** 1 Department of Clinical and Experimental Medicine, Pisa University, Via Savi 27, 56100 Pisa, Italy; 2 Department of Surgery, Medical, Molecular, and Critical Area Pathology, Pisa University, Pisa, Italy

**Keywords:** Empagliflozin, SGLT2, Mycardial dysfunction, GLS, Speckle-tracking, Cardiovascular

## Abstract

**Background:**

Asymptomatic left ventricular (LV) dysfunction is highly prevalent in type 2 diabetes patients. Unlike the other hypoglycemic drugs, SGLT2 inhibitors have shown potential benefits for reducing cardiovascular death and risk factors, aside from lowering plasma glucose levels. With this study we aim at determining whether the treatment with empagliflozin is associated with an improvement in LV functions in diabetic patients with asymptomatic LV dysfunction against Sitagliptin, which is presumably neutral on myocardial function. To determine changes in LV systolic and diastolic functions we will use s*peckle*-*tracking echocardiography*, a novel sensitive, non-invasive, bedside method allowing the calculation of LV global longitudinal strain (GLS), an index of myocardial deformability, as well as 3D echocardiography, which allows a better evaluation of LV volumes and mass.

**Methods:**

The EMPA-HEART trial will be a phase III, open label, active-controlled, parallel groups, single centre, exploratory study conducted in Pisa, Italy. A cohort of 75 diabetic patients with normal LV systolic (2D-Echo EF > 50%) and renal (eGFR sec MDRD > 60 ml/min/1.73 mq) functions and no evidence of valvular and/or ischemic heart disease will be randomized to either Empagliflozin 10 mg/die or Sitagliptin 100 mg/die. The primary outcome is to detect a change in GLS from baseline to 1 and 6 months after treatment initiation. The secondary outcomes include changes from baseline to 6 months in 3-D Echocardiography EF, left atrial volume and E/E′, VO_2_max as measured at cardiopulmonary test, cardiac autonomic function tests (R–R interval during Valsalva manoeuvre, deep-breathing, lying-to-standing), and the determination of a set of plasma biomarkers aimed at studying volume, inflammation, oxidative stress, matrix remodelling, myocyte strain and injury.

**Discussion:**

SGLT2 inhibitors might affect myocardial functions through mechanisms acting both directly and indirectly on the myocardium. The set of instrumental and biohumoral tests of our study might actually detect the presence and entity of empagliflozin beneficial effects on the myocardium and shed light on the mechanisms involved. Further, this study might eventually provide information to design a clinical strategy, based on echocardiography and/or biomarkers, to select the patients who might benefit more from this intervention.

*Trial registration* EUDRACT Code 2016-0022250-10

## Background

### Diabetes mellitus and myocardial dysfunction

The pathogenesis of myocardial dysfunction in type 2 diabetes mellitus (T2D) is multifactorial, being the diabetic myocardium chronically exposed to a mixture of metabolic, haemodynamic, macro and microvascular insults, which altogether are responsible for the greater incidence of heart failure (HF) and cardiovascular death of these patients [1]. The peculiar complex and multifactorial pathogenesis of HF in T2D patients fit into a disease-specific condition named *diabetic cardiomyopathy*, which may be found in isolation or associated to coronary artery and/or hypertensive heart disease [2, 3]. In this scenario, is not surprising that asymptomatic left ventricular (LV) dysfunction, defined as either systolic or diastolic abnormalities in the absence of clinically detectable cardiac disease, is highly prevalent in T2D patients, with estimates ranging from 50 to 70% [4, 5]. A better knowledge of its early-stage risk factors and its natural evolution would allow the design of effective prevention strategies as strongly recommended by current clinical guidelines, which emphasize the importance of early diagnosis and interventions in subjects at risk [3]. However, one major limitation to this approach is the lack of consensus with regard to the best method to detect asymptomatic LV dysfunction, which is responsible for the limited information, particularly about the effects of antidiabetic drugs on LV performance. The available methods for accurately measuring cardiac functions are either too complex (cardiac magnetic resonance) or not sensitive enough (brain-derived natriuretic peptide—BNP-, 2D ejection fraction—EF- and tissue Doppler) to detect subtle changes in cardiac performances, particularly in asymptomatic subjects.

### Sodium-glucose linked co-transporter 2 (SGLT2) inhibitors in cardiovascular disease

In the kidney, plasma glucose is freely filtered into the urine through the glomerulus and is reabsorbed in the tubule through an efficient system of ATP-dependent transporter proteins, the sodium-glucose-linked co-transporters (SGLT). SGLTs are found in the proximal tubule in two different isoforms, namely SGLT1 and SGLT2. Both proteins bind and transport sodium and glucose, SGLT2 in a one-to-one and SGLT1 in a two-to-one ratio, while showing some clinically relevant differences. SGLT2, a high-capacity, low-affinity transporter, is present in segment 1 of the tubule and normally accounts for almost 90% of the entire kidney glucose reuptake. In T2D, secondary to chronic hyperglycemia, the expression of SGLT2 is upregulated, leading to an increased renal tubular reabsorption exacerbating hyperglycemia [6]. Conversely, SGLT1 is a low-capacity, high-affinity transporter present in segment 3 of proximal tubule, but that is also expressed, to a greater extent than in the kidney, in the small intestine and in the heart [7]. In the latter, SGLT1 is thought to be localized both in cardiomyocytes and in capillaries [8]. Besides their proven efficacy in lowering plasma glucose levels, SGLT2 inhibitors have also been shown to have potential benefits for improving other cardiovascular risk factors, such as body weight and blood pressure, while being well tolerated [9]. The study EMPA-REG, quite unexpectedly, reported in the diabetic population a 35% reduction in hospitalization for HF and a 32% reduction in cardiovascular death largely due (data from the Supplementary appendix of ref [10]) to a reduction in fatal events related either to worsening of HF (incidence rates: 0.2 vs 0.8%) or to sudden death (incidence rates: 1.1 vs 1.6%, in the pooled empagliflozin and placebo group, respectively), while outcomes more closely related to atherosclerosis, e.g. cerebrovascular and ischemic heart diseases, were only marginally affected. In addition, the benefit was evident very early after treatment initiation (~ 6 months). Since other oral hypoglycemic drugs have been shown either to increase the risk of HF [11, 12], or to be neutral [13, 14], then the hypothesis that empagliflozin might directly and rapidly improve LV function, irrespective of the improvement in metabolic control, has been raised. Similar results on HF have been recently reported with another SGLT2 inhibitor, canagliflozin, in the recently published CANVAS study [15] and also in a large observational study on users of SGLT-2 inhibitors (CVD-REAL) in which the majority of the subjects were treated with dapagliflozin [16]. Diastolic function in ob/ob in a preliminary report in 10 patients of EMPA-HEART study short-term (151 days) with empagliflozin was associated with a 15% reduction in LV mass index and a 13% increase in early lateral annular tissue Doppler velocity (e′), a proxy of LV compliance. Finally, the recent article by Januzzi et al. [17] demonstrated that chronic therapy with Canagliflozin is associated with delayed physiologic rise in serum NT-proBNP and high sensitive troponin I (hsTnI) in a 2 years’-follow up in older T2D patients by a little but significative amount, thus suggesting a beneficial role. Despite this, information with respect to SGLT2 inhibitors effect on myocardial function is very limited.

### Speckle-tracking and 3D echocardiography for early detection of myocardial dysfunction

A recent sensitive, non-invasive and bedside method for the evaluation of myocardial systolic and diastolic function, namely 2D-*speckle*-*tracking echocardiography* (STE), has recently been developed and validated (against tissue Doppler, cardiac magnetic resonance and sonomicrometry) in large cohorts of patients, including subjects with T2D [18–21]. STE exploits the natural acoustic markers that are evident in grey-scale ultrasound images within the myocardial tissue, which corresponds to specific anatomical structures [22]. The spatial displacement of the speckles during the cardiac cycle reflects the strain of the myocardium and can be tracked on a frame-by-frame basis for each region of interest both in longitudinal and radial directions according the standard echocardiographic approaches. Strain values are calculated for each standard myocardial segment (segmental strain) and the average value of all segmental strains provides the global LV strain.

Currently only LV global longitudinal strain (GLS) has been validated. Interestingly, among T2D outpatients without valvular disease, previous cardiac events or symptoms, and with a normal 2D EF, an abnormal GLS measured by STE has been consistently found in 30–50% of the subjects [23–25]. Of note, in T2D patients the condition of normal EF and abnormal GLS was associated with a reduced VO_2_max at cardiopulmonary test [20], and, most importantly, to an adverse cardiovascular outcome at 10-year follow-up [26].

Very recently, 3D echocardiography has also become available for clinical use. Combined matrix array transductors allow contemporary and real-time bedside evaluation of 2D and 3D images. Being more accurate with respect to 2D echography, which relies on a set of approximate geometric assumptions, it currently represents a promising tool in the measurement of both systolic and diastolic volumes, as well as LV mass and remodeling itself [27].

### Biomarkers of cardiac function and mechanisms of disease

In order to detect subclinical LV dysfunction and to provide information on the mechanisms potentially implicated in the diabetic cardiac dysfunctions, we have chosen a set of plasma biomarkers, which not only look at specific pathways (volume, inflammation, oxidative stress, matrix remodelling, myocyte strain and injury) but were also validated against hard endpoints [28].

#### Volume biomarkers and total body water (TBW)

We will measure plasma aldosterone (DiaSorin, Saluggia, Italy; intra-assay 9.7% and inter-assay 11.5%, n.v. 3.5–30.0 ng/dl) and renin (CisBIO, Bedford, MA, USA; intra-assay 1.8% and interassay 4.0%, n.v. 5.1–59.4 pg/ml) as a proxy for rennin-angiotensin-aldosterone system (RAAS) activation, which track effective circulating volume. The activation of the RAAS system also has been shown to be a prognostic marker in patients with HF. In a cohort of 996 subjects [29] plasma renin has been shown to predict death at 3 years follow-up, independently of the most important predictors: age, EF and NT-pro-BNP. The body fluid status will be assessed also by measuring total body water (TBW) using the deuterated water dilution method [30]. A fixed amount (10 g) of 99% deuterated water (Sigma Aldrich) will be ingested at 10:00 p.m. and the patient will collect a urine sample the subsequent morning at 7:00. TBW will be calculated as the deuterium dilution space divided by 1.04, to correct for exchange of the deuterium label with non-aqueous H of body chemicals [31]. Isotope abundances in urine were determined in duplicate by nuclear magnetic resonance (^2^H NMR) according to the protocol described by Khaled et al. [32].

#### Myocardial parietal stress biomarkers

We will measure natriuretic peptides (NPs); namely brain natriuretic peptide (BNP) and N-terminal pro-brain natriuretic peptide (NT-proBNP) that are released into the bloodstream in direct proportion to the mechanical stress of the myocardium [33]. As such, NPs have been proved useful in screening asymptomatic subjects at risk of developing HF, such as the elderly and those with hypertension, diabetes, or asymptomatic coronary artery disease [33, 34], and will be used in this study for grading and monitoring the changes in asymptomatic LV dysfunctions observed with STE. More recently also proadrenomedullin has been validated as a myocardial parietal stress plasma biomarker. This cardiomyocyte-derived peptide is released in proportion to cardiac pressure and volume [35] and its serum levels are useful for grading HF severity and predict death, either alone or when combined with NT-proBNP [36].

#### Cardiomyocyte injury biomarkers

Plasma troponin-T will be measured to assess cardiomyocyte injury being present in HF patients not only as a result of ischemia, but also as a consequence of inflammation, oxidative stress, and neurohormonal activation. In fact, with the high-sensitive assay, abnormal troponin levels can be detected in up to 92% of HF patients, wherein are of high prognostic value being associated with an increased risk of death even after adjustment for baseline variables and BNP [37].

#### Inflammation/oxidative stress biomarkers

High-sensitivity C-reactive protein (hsCRP) is an acute-phase reactant synthesized by hepatocytes in response to the proinflammatory cytokine interleukin-6 (IL-6) [38] in a variety of inflammatory processes. Its serum levels appear to be positively associated with HF severity and progression being an independent predictor of adverse outcomes in patients with acute or chronic HF [39, 40]. Further, notwithstanding its non-specificity, CRP might have a causal role in vascular and/or myocardial damage, since it exerts direct adverse effects on the vascular endothelium by reducing nitric-oxide release and increasing endothelin-1 production, as well as by inducing expression of endothelial adhesion molecules [41]. Also tumor necrosis factor-alpha (TNF-α) has been proposed as a marker of HF and asymptomatic LV dysfunction. This pro-inflammatory cytokine has been observed to cause LV dilatation, seemingly by activating matrix metalloproteinases [42]. Despite the fact that the blockade of TNF-α did not result in clinical benefit in patients with HF [43], TNF-α levels have been then proposed to predict the future development of HF in asymptomatic elderly subjects [44]. Together with inflammation, also oxidative stress plays a role in determining myocardial damage. Since reactive oxygen species are many and are difficult to measure we focused on the plasma biomarkers that have been validated in clinical trials: myeloperoxidase (MPO). The levels of plasma MPO, in fact, are elevated in coronary artery disease, possibly having a role in determining and revealing LV remodeling [45]. Moreover, its levels correlate with the severity of HF and are independent predictors of death from HF, even after adjustment for baseline variables [46].

#### Matrix remodelling biomarkers

Being adverse myocardial remodelling largely dependent from excessive collagen synthesis and associated with impaired LV function and adverse clinical outcomes in patients with HF, we decided to measure type III pro-collagen, whose serum level has been shown to be an independent predictor of adverse outcomes in patients with HF [47].

## Methods

### Rationale and aim of the study

The specific medical need addressed by this study is to offer T2D patients, who appear to be at high risk for developing HF and its lethal consequences, a new therapeutic option capable not only of controlling their plasma glucose but also improving their underlying subclinical cardiomyopathy. Further, this study might eventually provide information to design a clinical strategy, based on echocardiography and/or biomarkers, to select the patients who might benefit more from this intervention.

With this study we aim at verifying whether, in T2D patients with subclinical cardiomyopathy (i.e. without: overt HF symptoms and signs, significant valvular heart disease, evidence of inducible myocardial ischemia and with 2-D ejection fraction ≥ 50%) the treatment with empagliflozin is associated with an improvement in LV systolic function, as measured by GLS through STE, in comparison to sitagliptin, an equally effective plasma glucose lowering agent, which has been shown to be neutral on HF-related events prevention.

### Study design

**Fig. 1:**
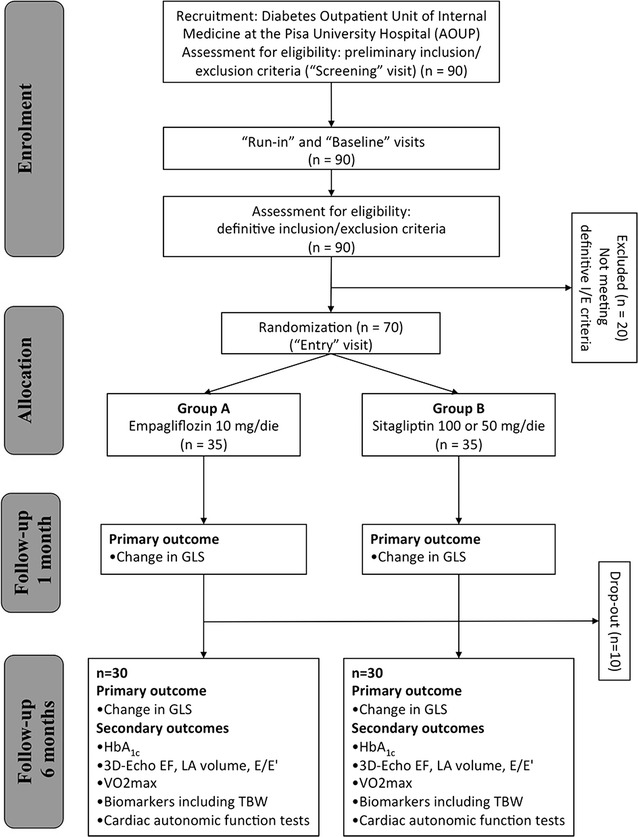
Study design flowchart. *EF* ejection fraction, *GLS* global longitudinal strain, *HbA*_*1c*_ glycated haemoglobin, *LA* left atrium, *TBW* total body water, *VO*_*2*_ *max* O_2_ consuption at peak of stress

The EMPA-HEART trial will be a phase III, open label, active-controlled, parallel groups, single centre, exploratory study conducted in Pisa, Italy (Fig. 1). This is a proof-of-concept study aiming at evaluating whether the chronic treatment with the SGLT-2 inhibitor empagliflozin has an effect on myocardial function above and beyond its effect on metabolic control. The patients will be randomized to either empagliflozin 10 mg/die or to Sitagliptin 100 mg/die as an active comparator, which has a similar potency in terms of glucose control and, on the bases of large clinical trials, does not influence the risk of developing HF. The study consists of 7 visits (see Table 1) and will last 2 years, therefore, being the start programmed for July 2017 the end of the study is expected for July 2019.

**Table 1:** Study conduction flowchart overview of all visit and tests scheduled for participants of the EMPA-HEART trial

Visits	Screening	Run-in	Baseline	Entry	1 Month	6 Months	Study end
Time (week)	*−* *5*	*−* *3*	*−* *1*	*0*	*4*	*24*	*26*
Medical history		X					X
Physical examination		X		X	X		X
ECG		X					X
Preliminary I/E criteria	X						
Definitive I/E criteria				X			
Informed consent	X						
Randomization				X			
Drug prescription				X			
Biomarkers		X					X
Body water assessment	X						X
Safety sample		X					X
Drug compliance							X
Adverse effect					X		X
Cardiac autonomic tests		X					X
Exercise test			X			X	
Echocardiography			X		X	X	

### Study population

Volunteers will be recruited among patients attending visits at the Diabetes Outpatient Unit of Internal Medicine at the Pisa University Hospital (AOUP). The experimental sessions will take place in the *Laboratory of Nutrition, Metabolism and Atherosclerosis* and part in the unit of *Cardio*-*angiology* of the AOUP under the responsibility of AN and VD, respectively. All potential participants who meet the following criteria will be eligible for the trial:

#### Inclusion criteria

- Male or female affected by type 2 diabetes mellitus (T2D).
- Age ≥ 40 and ≤ 80 years.
- HbA_1c_ levels ≥ 53 (7%) and ≤ 69 mmol/mol (8.5%).
- On stable (since 3 months) antidiabetic therapy with either Metformin alone or Metformin + basal insulin (this constraint is determined by the present Italian prescription rules).
- On stable (since 3 months) cardio-active therapies (e.g. anti-hypertensive drugs, diuretics, drugs for asthma or migraine prophylaxis).
- Preserved kidney function as defined by eGFR ≥ 45 ml min^−1 ^1.73 m^2^.
- Preserved left ventricular function as defined by EF ≥ 50%.

#### Exclusion criteria

- Refuse or inability to give informed consent.
- Patients unlikely to comply with the protocol or unable to understand the nature, scope and possible consequences of the study.
- Employees of the investigator or study centre (i.e. principal investigator, sub-investigator, study coordinators, other study staff, employees, or contractors of each), with direct involvement in the proposed study or other studies under the direction of that investigator and/or study centre, as well as family members of the employees or the investigator.
- Patients with type 1 diabetes mellitus.
- Pregnancy or active breast feeding.
- History of hospitalization for acute coronary syndrome or heart failure.
- Respiratory insufficiency or history of clinically significant respiratory diseases (chronic obstructive pulmonary disease).
- NYHA class III and IV or other symptoms of heart failure (breathlessness, orthopnoea, paroxysmal nocturnal dyspnoea, fatigue, increased time to recover after exercise, ankle swelling) unrelated to most common non-cardiac causes.

Signs of heart failure (elevated jugular venous pressure, third heart sound, laterally displaced apical impulse).

- Acute or chronic inflammatory diseases.
- History of active neoplastic disease within the last 5 years.
- Patients with known hypersensitivity to empagliflozin and its excipients.
- Volume-depleted patients or those who, in the judgement of the investigator, may be at risk for dehydration (abuse of diuretics or laxatives, chronic diarrhoea etc.).
- History of recurrent or serious genitor-urinary infections.
- Patients with known hypersensitivity to sitagliptin and its excipients.
- History of acute or chronic pancreatic disease.
- Patients who received any investigational new drug within the last 12 weeks.
- Severe obesity (body mass index, BMI ≥ 40 kg/m^2^).
- Uncontrolled blood pressure, defined as > 160/100 mmHg.
- Expected glomerular filtration rate (eGFR) < 45 ml/min/1.73 m^2^.
- Severe hepatic insufficiency and/or significant abnormal liver function defined as aspartate aminotransferase (AST) > 3× upper limit of normal (ULN) and/or alanine aminotransferase (ALT) > 3× ULN or total bilirubin > 2.0 mg/dl.
- Cardiac arrhythmia (2nd grade AV block, atrial fibrillation, pacemaker, high incidence premature beats).
- Clinically relevant cardiac valvular disease.
- Ejection fraction < 50% or presence of regional left ventricular contraction impairment.
- Poor acoustic window or poor quality of echocardiographic imaging.
- Inability to perform the cardiopulmonary exercise test.
- Evidence of inducible myocardial ischemia at the cardiopulmonary test.

### Randomisation and treatment allocation

Patients will be randomized to a 26-week treatment with either empagliflozin 10 mg or sitagliptin 100 mg/daily as add-on to the background therapy. This will be done with a randomization matrix of 75 numbers calculated using the web-based service at http://www.random.org, assigning pair and even numbers to empagliflozin and sitagliptin, respectively. If the patient requires additional treatment for the presence of hyperglycemia, either symptomatic or detected at self-glucose monitoring (fasting plasma glucose (≥ 10 mM), the following measures will be sequentially taken: (a) lifestyle measures reinforcement, (b) initiation or up titration of metformin, (c) initiation or up titration of basal insulin, (d) withdrawal from the trial.

### Study objectives

#### Primary objective

To verify whether, in our population of T2D patients with normal 2-D EF (≥ 50%) and without evidence of cardiac valvular or ischemic disease, the chronic treatment with empagliflozin is associated with an improvement in LV systolic function as measured by GLS through STE in comparison to sitagliptin, an equally effective plasma glucose lowering agent presumably neutral on cardiac function.

#### Secondary objectives

1. To compare the effects of the 2 treatments on:
  3D echo left ventricular systolic and diastolic functional parameters
  Plasma mechanism-oriented biomarkers
  VO_2_max
  Cardiac autonomic function tests.
2. To test whether the effect of empagliflozin differs in the subgroup of patients with more pronounced abnormalities in cardiac systolic function at baseline (GLS > − 18%).
3. To assess in the whole cohort the relationship between the changes in cardiac function indices (GLS or 3D) and the concomitant changes in cardiac biomarkers and/or in blood pressure, weight and degree of metabolic control (exploratory analysis on the mechanisms).

### Study endpoints

#### Primary outcome variable

Changes in global longitudinal strain (GLS) from baseline to 1 and 6 months after treatment initiation.

#### Secondary outcome variables

Changes from baseline at 6 months after treatment initiation in

1. 3-D Echocardiography EF, left atrial volume and E/E′.
2. VO_2_max, as measured at cardiopulmonary test.
3. Myocardial parietal stress plasma biomarkers (BNP, NT-proBNP, proadrenomedullin), inflammation/oxidative stress plasma biomarkers (hsCRP, TNF-alpha, mieloperoxidase, uric acid) and cardiac remodelling/cytolysis biomarkers (type III pro-collagen, troponine), body volume biomarkers (total body water, plasma renin and aldosterone).
4. Cardiac autonomic function tests (R–R interval during Valsalva manoeuvre, deep-breathing, lying-to-standing).

#### Exploratory pre-defined hypothesis-driven analyses

1. Whether changes from baseline at 6 months after treatment initiation in plasma markers of volume control (plasma aldosterone-to-renin ratio and total body water), cardiomyocyte strain (BNP, NT-proBNP, proadrenomedullin), inflammation/oxidative stress (hsCRP, TNF-α, myeloperoxydase, uric acid), matrix remodeling (procollagen type III) and myocyte injury (Troponin T) help understanding the mechanisms of action through which the treatments exert their effect/s on the heart.
2. Whether changes from baseline at 6 months after treatment initiation in cardiac autonomic function score (based on RR changes with Valsalva, deep breathing, standing-to-laying) contribute to the mechanisms of action of the treatments.
3. Whether the effects of the treatments differ in the subgroup of patients whom at baseline have mild abnormalities in cardiac systolic function or abnormal values of plasma biomarkers or abnormal cardiac autonomic function tests [lower 50 percentile of GLS, atrial enlargement (LAVI > 34 ml/m^2^), LV hypertrophy (LVMI > 115 or 90 g/m2, males and females, respectively), diastolic dysfunction (E/e′ ≥ 13) and myocardial strain (Nt-pro-BNP > 125 pg/ml)].
4. Whether the changes in cardiac function are dependent on the concomitant changes in blood pressure, body weight and/or the improvement of the metabolic control and/or fasting plasma ß-OH butirrate concentration.

### Power calculation and sample size

The size of a change in GLS that is considered clinical relevant is based on the following data: (a) mean GLS (± SD) as assessed in a population of 114 type 2 diabetic subjects without evidence of cardiac disease and with 2D-EF ≥ 55% according to (15) was: − 19 (± 3) %; (b) a difference in GLS of 2.5% (− 21.2 ± 2.7 vs − 18.7 ± 2.8%) was observed between stage A and stage B heart failure in non-diabetic patients [20], and (c) a similar difference is present between subjects with and without diabetes [48]. A difference ≥ 2.5% is therefore considered highly clinically meaningful. Given the nature of the study (within subject repeated measures) a difference ≥ 1.5% would be considered relevant from a pathophysiologic point of view. Alpha error is always kept at 0.05. Estimation of the SD of the difference is based on an observational study performed in 112 type 2 diabetic patients with normal EF in whom after 2 years the GLS changed from − 19.7 ± 4.0 to − 18.9 ± 3.8% a difference of 0.8%, which was statistically significant (p < 0.001) at a paired t-test. The SD of the difference in GLS is not reported, however it can be estimated using a web-based engine (http://www.biomath.info/power/prt.htm) on the bases of the available mean and SD and by attributing a value to the correlation between the two repeated measurements of 0.80 on the bases of the intra observed error in GLS measurement 0.7 ± 0.4% according to Kosmala et al. [20] and on personal data. This data allowed the SD to be estimated as 2.5%.

Therefore, to detect an absolute ≥ 2.5% difference in GLS, a sample size of 30 subjects is required per arm. Considering a 21% drop-out, sample size will be 75 recruited subjects, to have 60 completed. In terms of Power for a total sample size of 60 (2 groups of 30), given the above-mentioned figures in a paired design (either t-test or Wilcoxon) within each of the two treatment groups (n = 30) the power to detect a difference of 2.5 and 1.7% is 0.99 and 0.95, respectively. In an MANOVA design for three repeated measures (baseline, 1, 6 months) the power of the study to detect a difference between the treatments of 2.5 or 1.7% is 0.98 and 0.80, respectively. In an ANCOVA design with 4 covariates a sample size of 60 subjects will have the power of 0.71 to detect an effect of 0.10 (expressed as of proportion of variance explained by the effect under consideration), which is commonly considered small in biostatistics and a power of 0.99 to detect a medium effect (0.25). The analysis will be 2-sided, PPS, no interim. 

## Discussion

**Fig. 2:**
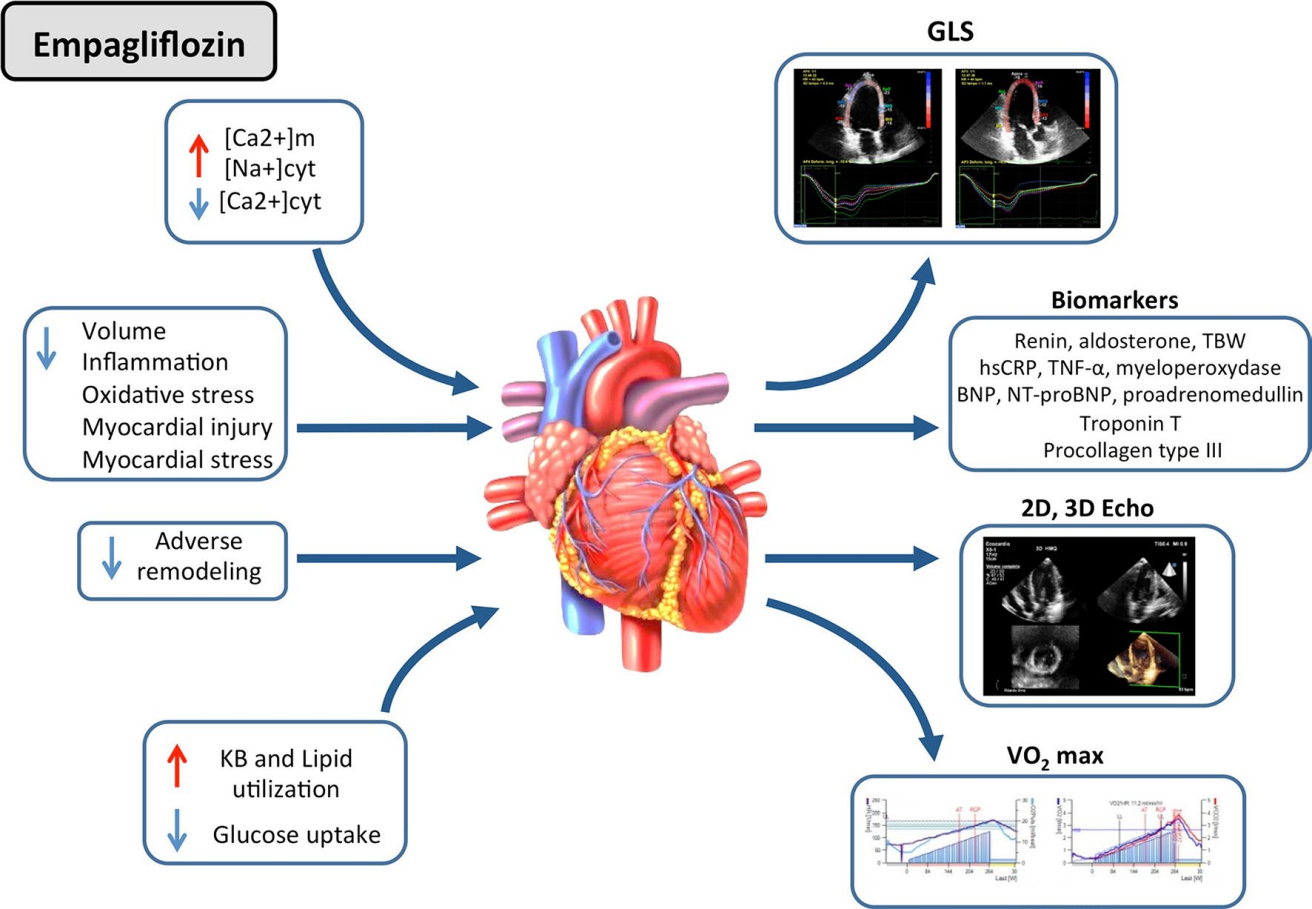
Potential mechanisms implicated in empagliflozin’s beneficial effects on the myocardium (on the left) and the biohuoral/instrumental tests performed to highlight them (on the right of the figure). The red vertical arrows indicate an increment, the blue vertical ones indicate a decrement. *cyt* cytoplasm, *GLS* global longitudinal strain, *hsCRP* high-sensitive C-reactive protein, *KB* ketone bodies, *m* mitochondrion, *TBW* total body water, *TNF-α* tumor necrosis factor-alpha, *VO*_*2*_ *max* O_2_ consuption at peak of stress

With this study we aim at demonstrating whether the treatment with empagliflozin is associated with the reversal of the earliest signs of diabetic systolic and diastolic dysfunctions, and at shedding light on the underlying mechanism/s involved (Fig. 2).

The reasons for concentrating on the earliest stages of HF stems from the common idea that this condition is more susceptible to reversal and also from the data of the EMPAREG OUTCOME trial showing the more robust effects, both in terms of hospitalization for HF and cardiovascular death, in the patients without HF at baseline or diagnosed during the study [10]. We therefore focused on patients with normal EF without current or prior symptoms of overt HF, but at high risk for the condition for presence of T2D and allowing for arterial hypertension and structural (LV hypertrophy) or functional (diastolic dysfunction) or biochemical (NT-pro-BNP) evidence of initial myocardial disease (Stage A and B HF according to ACC/AHA guidelines). In terms of pathophysiological changes the data are essentially based on few experimental studies and speculations [49]. SGLT2 inhibitors might actually affect myocardial functions through mechanisms that can act both directly and indirectly on the myocardium. Chronic treatment with SGLT2 inhibitors has been associated to a small blood pressure reduction (3–4 mmHg mean blood pressure) also confirmed by 24-h blood pressure monitoring [50], which was not associated to an increase in heart rate [51] and was independent of the number and type of baseline anti-hypertensive drugs [52]. The reasons for this blood pressure reduction, though not fully understood, is likely to involve the small (5%) weight reduction and the minor diuretic and natriuretic effects [50, 51] that are induced by glycosuria. Given the size of these changes, however, it is unlikely that they entirely justify the clinical outcomes. Since SGLT2 inhibitors reduce arterial blood pressure without a compensatory increase in heart rate, a reduction in the sympathetic tone [53] has also been suggested as a potential mechanism. Owing to its renal and hemodynamic effects, also other neurohormonal axes might be involved, particularly the RAAS and NPs, resulting in a change in TBW. To date, however, at the best of our knowledge no data is available. With regard to body fluid control the information is limited to a single preliminary study in which chronic treatment with dapagliflozin resulted in a 5% reduction in plasma volume [50]. A direct vascular effect might also contribute to blood pressure changes. Interestingly, a 8-week empagliflozin treatment reduced arterial stiffness in patients with type 1 diabetes [54]. Preliminary experimental data suggest that SGLT2 inhibitors may improve the vascular structural properties, interfering with collagen, elastin, advanced glycation end-products [55]. Moreover, the SGLT2 inhibitors-induced increased ketone bodies has been proposed to facilitate myocardial energetics [56]. In fact, myocardial utilization of β-OH-butirrate results in a significant increase in ATP production with respect to glucose and fatty acid oxidation and in an improved efficiency (hydraulic work/energy from O_2_ consumed) in a model of isolated working heart by 25% [57].

Together with the above-mentioned indirect effects, empagliflozin shows some direct myocardial effects as well. In cardiomyocytes, mitochondrial Ca^2+^ is considered to be one major activator of ATP synthesis and of the antioxidant enzymatic network [58]. Elevated cardiac cytoplasmic Na^+^ and Ca^2+^ concentrations and decreased mitochondrial Ca^2+^ concentration are characteristic hyperglycemia-induced drivers of HF and cardiac death. In a recent paper [59] empagliflozin was demonstrated to reduce cardiomyocytes cardiac cytoplasmic Na^+^ and Ca^2+^ concentrations and to increase mitochondrial Ca^2+^ levels by directly inhibiting Na^+^/H^+^ exchanger (NHE). Previous studies have demonstrated that chronic inhibition of NHE prevents or mitigates HF in animal models [60], and this might be one of the mechanisms through which empagliflozin protects from HF deaths and hospitalizations. Finally, recent observation raised the possibility that SGLT2 inhibitor treatment might also have a beneficial effect on cardiac remodeling. In a rat model of progressive HF, it has been observed that SGLT2 inhibition could attenuate the increase in LV mass and LV end-diastolic diameter [61]. In obese T2D (db/db) mice, a 10 week-long treatment with empagliflozin has been observed to reduce interstitial cardiac fibrosis, peri-coronary arterial fibrosis, coronary arterial thickening, cardiac interstitial macrophage infiltration, and cardiac superoxide levels [62]. In the same study, empagliflozin also ameliorated endothelial dysfunction. Moreover, in animal models, SGLT-2 inhibitors have been shown to reduce myocardial leukocytosis induced by hyperglycemia [63] and to reduce inflammation and oxidative stress [64] as well as possibly fibrosis [65]. The only study that is available on cardiac function is a preliminary observation done in 10 T2D patients by using standard 2D echocardiography, that reported a small improvement in LV mass (reverse remodeling) and diastolic function after 5 months of treatment with empagliflozin [21]. To date, no data on the effect of SGLT2 inhibitors on cardiac remodeling in subjects with HF is available, but the REFORM trial [66] is currently underway in order to investigate, in patients with T2D and HF, the effects of dapagliflozin on LV remodeling through cardiac magnetic resonance and cardiopulmonary stress test.

## Limitations

Despite the estimate high power of the study, this is an open, short-term, and relatively small trial. In order to prevent any influence of the researcher expectations (type 1 error) we will take care that the cardiologist performing the primary and several of the secondary outcome variables measurements are blind with respect to the treatment allocation. In addition, given the expected mild degree of systolic dysfunction at baseline caused by our strict selection criteria we are somehow protected from type 1 error although the risk to occur in a type 2 error is increased. However, by measuring a full set of other relevant myocardial function parameters and biomarkers we will actually be able to generate solid data-driven hypotheses for future studies. The recent evidence of the PROLOGUE trial (Yamada), showing that sitagliptin is able to prevent the time-related deterioration of diastolic function, raises the possibility that our comparator is not completely neutral on cardiac function. However, this effect was evident after 12, but not after 6 months of treatment and sitagliptin has been shown to be neutral on systolic function in patients with post-ischemic heart failure with mildly reduced ejection fraction [67]. Therefore, there will be, if any, a small risk of type 2 error, which we consider acceptable and eventually would, in case, reinforce any observed positive outcome of empagliflozin.

## Abbreviations

BMI: body mass index
BNP: brain-derived natriuretic peptide
EF: ejection fraction
eGFR: expected glomerular filtration rate
GLS: global longitudinal strain
HF: heart failure
hsCRP: high-sensitive C-reactive protein
LV: left ventricle
MPO: myeloperoxidase
NHE: Na^+^/H^+^ exchanger
NPs: natriuretic peptides
NT-proBNP: N-terminal-proBNP
RAAS: renin-angiotensin-aldosterone system
SGLT2: sodium-glucose cotransporter 2
STE: speckle-tracking echocardiography
T2D: type 2 diabetes
TBW: total body water
TNF-α: tumor necrosis factor-alpha
VO_2_max: maximal oxygen volume
